# The impact of astrocytic NF-κB on healthy and Alzheimer’s disease brains

**DOI:** 10.1038/s41598-024-65248-1

**Published:** 2024-06-21

**Authors:** Tee Jong Huat, Judith Camats-Perna, Estella A. Newcombe, Tessa Onraet, Daniel Campbell, Josiah T. Sucic, Alessandra Martini, Stefânia Forner, Mehdi Mirzaei, Wayne Poon, Frank M. LaFerla, Rodrigo Medeiros

**Affiliations:** 1 Clem Jones Centre for Ageing Dementia Research, Queensland Brain Institute, The University of Queensland, Brisbane, QLD Australia; 2 Centre for Stem Cell Ageing and Regenerative Engineering, The University of Queensland, Brisbane, QLD Australia; 3 Institute for Memory Impairments and Neurological Disorders, University of California, Irvine, 3400A Biological Sciences III, Irvine, CA 92697-4545 USA; 4 Clinical Medicine Department, Faculty of Medicine, Health and Human Sciences, Macquarie University, Sydney, NSW Australia; 5 Department of Neurobiology and Behavior, University of California, Irvine, Irvine, CA USA

**Keywords:** Alzheimer's disease, Astrocyte

## Abstract

Astrocytes play a role in healthy cognitive function and Alzheimer’s disease (AD). The transcriptional factor nuclear factor-κB (NF-κB) drives astrocyte diversity, but the mechanisms are not fully understood. By combining studies in human brains and animal models and selectively manipulating NF-κB function in astrocytes, we deepened the understanding of the role of astrocytic NF-κB in brain health and AD. In silico analysis of bulk and cell-specific transcriptomic data revealed the association of NF-κB and astrocytes in AD. Confocal studies validated the higher level of p50 NF-κB and phosphorylated-p65 NF-κB in glial fibrillary acidic protein (GFAP)^+^-astrocytes in AD versus non-AD subjects. In the healthy mouse brain, chronic activation of astrocytic NF-κB disturbed the proteomic milieu, causing a loss of mitochondrial-associated proteins and the rise of inflammatory-related proteins. Sustained NF-κB signaling also led to microglial reactivity, production of pro-inflammatory mediators, and buildup of senescence-related protein p16^INK4A^ in neurons. However, in an AD mouse model, NF-κB inhibition accelerated β-amyloid and tau accumulation. Molecular biology studies revealed that astrocytic NF-κB activation drives the increase in GFAP and inflammatory proteins and aquaporin-4, a glymphatic system protein that assists in mitigating AD. Our investigation uncovered fundamental mechanisms by which NF-κB enables astrocytes' neuroprotective and neurotoxic responses in the brain.

## Introduction

Astrocytes are a diverse type of glial cell with remarkable plasticity and play a critical role in maintaining the overall health and function of the central nervous system (CNS)^1,2^. They are involved in many complex processes, such as regulating the extracellular environment, providing metabolic support to neurons, mediating synapse formation and plasticity, modulating neural activity, and coordinating inflammatory responses. The ability of astrocytes to respond to various stimuli and adjust their activity accordingly is pivotal in counterbalancing disease states, including Alzheimer's disease (AD)^3,4^. In AD, astrocytes react functionally and morphologically to the neuropathological markers of the disease. Their signaling cascades enable essential neuroprotective outcomes such as the clearance of excessive neurotransmitters and toxic proteinaceous forms of β-amyloid (Aβ) and tau^5–9^. These cascades also facilitate efficient intercellular communication, allowing cells to react appropriately to microenvironment changes^10–12^. Failure of astrocytic functions due to genetic polymorphisms and cellular pathway dysregulation can significantly impact AD severity and trajectory^5,13–18^.

The nuclear factor-κB (NF-κB) family encompasses five distinct DNA-binding proteins that combine to form a diverse array of homodimers and heterodimers. This transcriptional factor is typically inactive in the cytoplasm of resting cells. Its canonical activation relies on the degradation of inhibitor of NF-κB (IκB) proteins, which occurs after they are phosphorylated by the IκB kinase (IKK) complex. Once within the nucleus, phosphorylation plays a role in further modulating the transcriptional functions of NF-κB. While each NF-κB dimer likely has unique regulatory functions, many target genes are common to several, if not all, NF-κB proteins. Functionally, NF-κB-regulated genes are predominantly immunoregulatory and inflammatory, anti-apoptotic, and genes that positively regulate cell proliferation^19^. NF-κB also mediates the expression of its inhibitory proteins, including A20, also known as tumor necrosis factor alpha-induced protein 3 (TNFAIP3), which inhibits and resolves the NF-κB-induced responses^20^. This transcriptional factor has also been designated as one of the likely regulators of the pro-inflammatory signature acquired by astrocytes^21,22^. Notably, dysregulated activation of NF-κB in astrocytes triggers numerous neurodegenerative pathologies^23–29^.

In the context of AD, astrocytic NF-κB is responsible for promoting the expression of multiple disease-relevant molecules, such as aquaporin-4 (AQP4)^7,30–32^, YKL-40 (also known as chitinase 3-like protein 1)^33,34^, interleukin-6 (IL6)^21,35,36^, nerve growth factor (NGF)^37,38^, tumor necrosis factor-α (TNFα)^39–41^, inducible nitric oxide synthase (iNOS)^40,42,43^, glutamate transporter-1 (GLT1)^44–47^, cyclooxygenase-2 (COX2)^48–50^, and apolipoprotein E (ApoE)^13–16,51^. NF-κB activation in astrocytes seems to have detrimental and beneficial effects on AD. Astrocytes rely on NF-κB to produce complement C3, which has been shown to negatively impact the morphology and function of neurons^52^. Deletion of the upstream activator of NF-κB, myeloid differentiation primary response 88 (MyD88), from astrocytes reduced pro-inflammatory responses, synaptic toxicity, and cognitive impairment caused by Aβ^53^. Conversely, astrocytic NF-κB helped clear Aβ through a process linked with the microglia polarization^54^. These findings suggest a complex role for astrocytic NF-κB in AD and underscore the need for further investigation into its mechanisms and effects. Hence, we evaluated the consequences of sustained activation of astrocytic NF-κB in the brain and how its inhibition affects the progression of AD-like pathology.

## Materials and methods

### Human databases

We obtained the normalized transcriptomic data from the human temporal cortex (TCX) in the MayoPilot RNAseq study, available in the AD Knowledge Portal (ID: syn5550404)^55^. The data comprises information from 78 non-AD subjects (37 female and 41 male) and 82 AD subjects (49 female and 33 male). All AD cases had a definite diagnosis of AD according to the National Institute of Neurological and Communicative Diseases (NINCDS) and Alzheimer’s Disease and Related Disorders Association (ADRDA) criteria and had a Braak neurofibrillary tangle (NFT) stage of IV or higher. Non-AD subjects had a Braak NFT stage of III or less and neuritic and cortical plaque densities of 0 (none) or 1 (sparse), according to the Consortium to Establish a Registry for Alzheimer’s Disease (CERAD). AD and non-AD subjects lacked any other major neurodegenerative disorder.

We accessed the levels of 250 immune-related genes, including transcriptional factors, cytokines, chemokines, and cell markers (Supplementary Table 1). We calculated the differential gene expression employing an unpaired t-test followed by Holm-Sidak’s multiple comparisons tests using Prism GraphPad Software (San Diego, CA, USA). The accepted significance level for the tests was *P* < 0.05. The volcano plots display the differences in Z-scores between AD and non-AD subjects. We performed the interaction networks functional enrichment analysis of the differentially expressed genes in the STRING database^56^. The association between NFKB1 and glial fibrillary acidic protein gene (GFAP), aldehyde dehydrogenase-1 family member L1 (ALDH1L1) or C-X3-C motif chemokine receptor-1 (CX3CR1) was determined using linear regression and Pearson correlation using Prism GraphPad Software.

Cell-specific NFKB1 transcriptomic data, including the statistical significance, were obtained from Mathys and colleagues^57^ and Sadick and colleagues^58^. Mathys’ study performed single-cell RNAseq in cells obtained from individuals with no pathology (N, individuals with no or very low Aβ burden or other pathologies, n = 24), early-AD (EAD, individuals with Aβ burden, but modest neurofibrillary tangles and modest cognitive impairment, n = 15), and late-AD (LAD, individuals with high Aβ burden, increased neurofibrillary tangles, global pathology, and cognitive impairment, n = 9). We represented the data relative to the mean level of the no pathology group. Sadick’s study employed isolated astrocytes from de-identified human post-mortem prefrontal cortex samples from AD (n = 9) and age-matched non-symptomatic (NS) patients (n = 5).

### Postmortem human brain

The Alzheimer’s Disease Research Center at the University of California, Irvine (UCI) provided age- and sex-matched TCX tissue from non-AD and AD individuals (n = 3–5). The UCI Institutional Review Board approved the protocol for securing postmortem brains, which followed the United States Federal Policy for the Protection of Human Subjects (UCI IRB HS# 2014–1526). The University of Queensland Human Research Ethics Committee approved our postmortem study (2017000490). Data were analyzed through an unpaired t-test with a confidence level of 95% using Prism (GraphPad Software). The accepted level of significance for the tests was *P* < 0.05.

### Animals

Experimental procedures used in the present study followed the Principles of Laboratory Animal Care from the National Institutes of Health (Bethesda, MD, USA) and the ARRIVE guidelines. The UCI Institutional Animal Care and Use Committee (AUP-20-087) and The University of Queensland Animal Ethics Committee (QBI/487/16) approved our study.

We generated mice with the tamoxifen-inducible IKK2 constitutively activated (IKK2CA) transgene in astrocytes (ALDH1L1-Cre-ERT-IKK2CA) from B6.Cg-Gt(ROSA)26Sor^tm4 (Ikbkb)Rsky^/J (IMSR_JAX: 008,242)^59^ and B6;FVB-Tg(Aldh1l1-cre/ERT2)1Khakh/J (IMSR_JAX: 029,655)^60^ mice. We also used female homozygous 3xTgAD mice harboring the APP Swedish, tau P301L, and presenilin-1 M146V mutations (MMRRC_034830-JAX) and the B6.129 non-transgenic (nTg) mice. We did not use male 3xTgAD mice, as they no longer display meaningful AD-like pathology^61^.

Animals were housed in a pathogen-free animal facility at controlled room temperature (22 ± 2 °C) and humidity (60–80%) under a 12:12-h light–dark cycle (lights on at 6 AM) with ad libitum access to food and water. All mouse strains are available through The Jackson Laboratory (Bar Harbor, ME, USA).

### Tamoxifen treatment

5-week-old ALDH1L1-Cre-ERT-IKK2CA mice were injected intraperitoneally (IP) with tamoxifen (75 mg/Kg) or vehicle (corn oil) once daily for five consecutive days. Each group consisted of four female mice and one male mouse due to availability with correct genotype. Brain samples were collected approximately three months after tamoxifen injection.

### Adeno-associated virus (AAV) stereotactic injection

Sixteen-month-old nTg (n = 3 per group) and 3xTgAD (n = 4–6 per group) mice were anesthetized using isoflurane and secured on a stereotaxic apparatus (Stoelting, Wood Dale, IL, USA). We administered 1.44E10 genome copies of AAV5-GFAP-m-A20-P2A-YFP (A20, Vector Biolabs) and AAV5-CAG-GFP (CTRL, Vector Biolabs) into both sides of the hippocampus at the coordinates: anterior–posterior − 2.06 mm; dorsoventral − 1.95 mm; mediolateral ± 1.75 mm. The injection site was held for 5 min, and the animals were allowed to recover before returning to their home cages. Brain samples were collected approximately three months post-AAV injection.

### Mouse brain dissection

Mice were deeply anesthetized with sodium pentobarbital (350 mg/Kg, IP) and sacrificed by transcardial perfusion with ice-cold phosphate-buffered saline (PBS). Brains were extracted and sliced in half through the sagittal plane. The hippocampus and cortex were dissected from the left hemisphere, snap-frozen in liquid nitrogen, and stored at -80 °C. The right hemisphere was fixed in 4% paraformaldehyde in PBS at 4 °C for 48 h and then stored in 0.02% sodium azide in PBS at 4 °C.

### Brain slicing

Fixed brain tissues were cryoprotected in 30% sucrose solution in PBS at 4 °C. Frozen human and mouse brain tissues were sectioned into 20 and 40 μm sections using a Leica SM2010R freezing microtome (Leica Microsystems, Bannockburn, IL, USA). Brain sections were stored in 0.02% sodium azide in PBS at 4 °C.

### Immunofluorescence

Free-floating sections were incubated with 2% bovine serum albumin and 0.1% Triton X-100 in tris-buffered saline (TBSB) for 1 h. Using the same buffer solution, sections were incubated overnight at 4 °C with the following primary antibodies: GFAP, p50 NF-κB, neuronal nuclei (NeuN), microtubule-associated protein 2 (MAP2), p16^INK4A^ (P16), A20, AQP4, , phosphorylated-p65 (p-p65) NF-κB, cluster of differentiation 68 (CD68) (Abcam), ionized calcium-binding adapter molecule 1 (IBA1) (FUJIFILM Wako Chemicals, Osaka, JP), 6E10 (BioLegend, San Diego, CA, USA), phosphorylated-p50 (p-p50) NF-κB, AT8 (Thermo Scientific, Waltham, MA, USA), and tau (Agilent, Santa Clara, CA, USA). As a blocking buffer, we incubated samples in 3% normal serum in TBSB for 1 h. Sections were then rinsed and incubated for 1 h with secondary Alexa Fluor-conjugated antibodies (Invitrogen, Carlsbad, CA, USA). Finally, brain sections were mounted onto gelatin-coated slides in Fluoromount-G (Southern Biotech Associates, Birmingham, AL, USA). The specificity of the immune reactions was controlled by omitting the primary antibody.

Confocal images were acquired via sequential scanning using a Leica DM2500 confocal laser microscope and Leica Application Suite Advanced Fluorescence software (Leica Microsystems). Five to ten three-dimensional (3D) confocal images were acquired per human subject. In mice, immunostaining was assessed at comparable hippocampal coronal sections positioned between 1.34 mm and 2.54 mm posterior to the bregma. As a minimum, three 3D confocal images were acquired per mouse. Using the colocalization and surface tools, quantitative volumetric analysis of the 3D images was performed with the Imaris software (Bitplane, Inc., South Windsor, CT, USA).

### Brain lysates

Frozen tissues were ground into powder in liquid nitrogen and lysed in ice-cold T-PER extraction buffer (Thermo Scientific) containing phosphatase inhibitor cocktail 2 (Roche, Basel, CH) and complete Mini EDTA-Free protease inhibitor cocktail (Sigma Aldrich, St. Louis, MO, USA). Samples were lysed in four cycles of 15 s and 5800 rpm at 4 °C, with 45 s intervals, using the Precellys homogenizer (Bertin, Montigny-le-Bretonneux, FRA). Samples were kept on ice for 10 min before tissue lysates were separated by centrifugation at 100,000 × g for 60 min at 4 °C to obtain the T-PER soluble fraction. The resulting pellet was resuspended and homogenized in 70% formic acid, followed by ultracentrifugation at 100,000 × g for 60 min at 4 °C. The supernatant was collected as the T-PER insoluble fraction. Insoluble fractions were diluted 1:20 in a neutralization buffer (1 mol/L Tris base and 0.5 mol/L NaH_2_PO_4_) before use. Protein concentrations were determined using the Bradford assay (Bio-Rad Laboratories, Hercules, CA, USA).

### Western blot

Proteins were separated on a Mini-Protean TGX 4–20% denaturing gel and transferred onto a nitrocellulose membrane (Bio-Rad Laboratories). Membranes were incubated in tris-buffered saline (TBS) containing 0.1% Tween-20 and 5% BSA for 1 h, followed by overnight incubation in the primary antibody at 4 °C. The following primary antibodies were used: anti-amyloid precursor protein (APP) C-Terminal (751–770) (Sigma Aldrich) and anti-glyceraldehyde-3-phosphate dehydrogenase (GAPDH) (Abcam). After three washes in TBS (5 min each), membranes were incubated with horseradish peroxidase (HRP)-conjugated or IRDye-conjugated secondary antibodies for 1 h (LI-COR Biosciences, Lincoln, NE, USA). The chemiluminescence signal was developed with SuperSignal West Dura (Thermo Scientific). Images were acquired using the Odyssey Fc imaging system and analyzed using the Image Studio software (LI-COR Biosciences).

### Capillary-based immunoassay

The automated capillary-based immunoassay was performed using the 12–230 kDa separation capillary kit and chemiluminescence modules (ProteinSimple, San Jose, CA, USA). Equal amounts of proteins from mice hippocampal lysates were loaded into the capillaries. The following primary antibodies were used: p65 NF-κB (Cell Signaling), p-p65 NF-κB, AQP4, and GAPDH (Abcam). Experiments were conducted according to the manufacturer’s instructions in the Jess system, and the chemiluminescence signal was determined in the Compass software (ProteinSimple).

### Proteomic analysis by mass spectrometry

This study's tandem mass tag (TMT) proteomics method follows our established protocols^53,62^. Raw data files were searched against protein sequence databases using Proteome Discoverer (version 2.1, Thermo Scientific). Data were processed using SequestHT and Mascot (Matrix Science, London, UK) search engines against all *Mus musculus* sequences downloaded from the SwissProt database (2018). The parameters for the data processing were as follows: enzyme: trypsin; maximum missed cleavages: 2; precursor mass tolerance: 20 ppm; fragment mass tolerance: 0.02 Da; dynamic modifications: oxidation (M), deamidated (N, Q), PyroGlu (Q), acetyl (protein N-terminus), acetyl protein N-term (Sequest), TMT6plex (K) and TMT6plex (N-term); static modification: carbamidomethyl (C); false discovery rate (FDR) and result display filters: protein, peptide, and peptide-spectrum matches (PSM) FDR < 1%, master proteins only. Differentially expressed proteins (*P* ≤ 0.05 and fold change ≥ 1.1) were analyzed using the Gene Set Enrichment Analysis (GSEA) software from the University of California, San Diego, and Broad Institute^63,64^ to determine gene ontology and cellular pathways terms.

### Electrochemiluminescence-linked immunoassay

Human Aβ levels were acquired using the V-PLEX Aβ Peptide Panel 1 (6E10). The Phospho(Thr^231^)/Total Tau Kit obtained total human tau and phosphorylated-tau levels. Levels of murine interferon-γ (IFNγ), IL-1β, IL-2, IL-4, IL-5, IL-6, IL-10, IL-12p70, keratinocyte chemoattractant (KC), and TNFα were determined using the V-PLEX Proinflammatory Panel 1 Mouse Kit. Equal amounts of proteins from mice brain lysates were loaded into the plates. Experiments were conducted according to the manufacturer’s instructions and read using the Sector Imager plate reader MSD MESO QuickPlex SQ 120 (Meso Scale Discovery, Rockville, MD).

### Statistical analysis

Data were presented as mean ± SD. Statistical evaluation of mice data was performed using an unpaired t-test with a confidence level of 95% using Prism (GraphPad Software). The accepted level of significance for the tests was *P* < 0.05. *P < 0.05; **P < 0.01; ***P < 0.001; ****P < 0.0001.

## Results

### NF-κB is coupled with clustered astrocytes in AD

**Figure 1:**
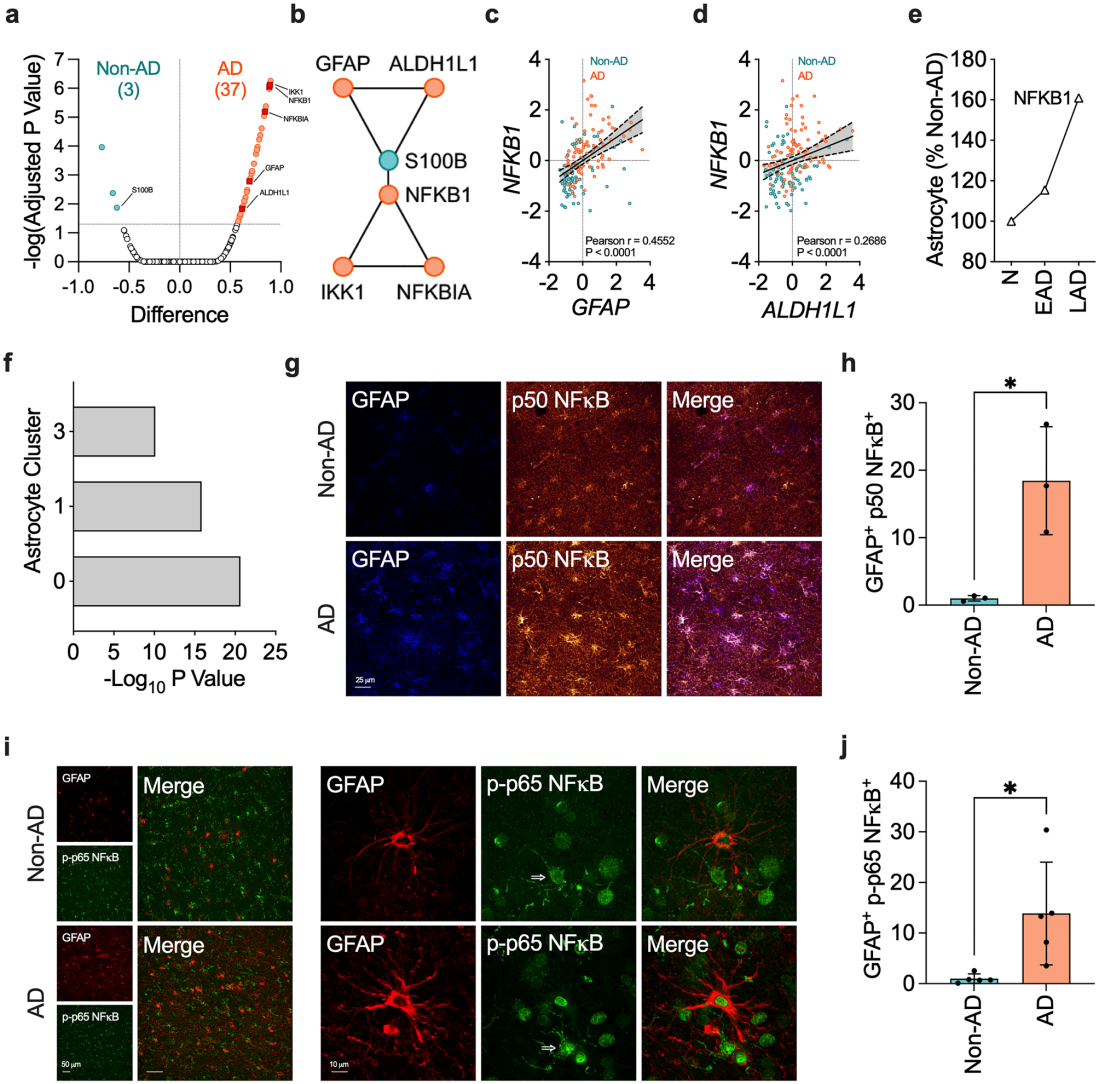
NF-κB is associated with astrocytes in AD. (**a**) Normalized transcriptomic data from the human temporal cortex (TCX, non-AD = 78, AD = 82) was obtained from the MayoPilot RNAseq study^55^. Differentially expressed genes were determined by unpaired t-test followed by Holm-Sidak’s multiple comparisons tests. The accepted level of significance for the tests was *P* < 0.05. The volcano plot displays the differences in Z-scores between AD and non-AD subjects. (**b**) Interaction networks functional enrichment analysis of the differentially expressed genes in the STRING database revealed a connection between the NF-κB signaling pathway and astrocytes. (**c**, **d**) The association between NFKB1 and (**c**) GFAP or (d) ALDH1L1 was determined using linear regression and Pearson correlation. (**e**) NFKB1 levels in astrocytes isolated from individuals with no AD pathology (N, n = 24), mid-level AD pathology (EAD, n = 15), and high-level AD pathology (LAD, n = 9) obtained from Mathys’ study^57^. We represented the data relative to the mean level of the no pathology group. (**f**) Significance thresholds of NFKB1 expression in the distinct astrocytes clusters obtained from Sadick’s study^19^. (**g**) Illustrative images of staining patterns and (**h**) volumetric analysis of p50 NF-κB colocalization in GFAP^+^-astrocytes in the TCX of non-AD and AD individuals (n = 3 per group, ten 3D images per group). (**i**) Illustrative images of staining patterns and (**j**) volumetric analysis of p-p65 NF-κB colocalization in GFAP^+^-astrocytes in the TCX of non-AD and AD individuals (n = 5 per group, five 3D images per group). Data were analyzed through an unpaired t-test. *P < 0.05. The arrow in the illustration panel indicates the presence of p-p65 NF-κB staining in a microglia-like shape.

We examined the levels of 250 genes in the MayoPilot RNAseq dataset^55^ to identify cellular and molecular processes associated with chronic inflammation in AD. Among cytokines, chemokines, enzymes, protein kinases, transcriptional factors, and cell markers, we identified 40 differentially expressed genes in the TCX of AD versus non-AD subjects (Fig. 1a, Supplementary Table 1). Our analysis also revealed that female subjects exhibited more significantly altered genes than male subjects (Supplementary Fig. 1a,b). This finding suggests a more pronounced inflammatory signature in the brains of female subjects.

In silico analysis in the STRING database^56^ highlighted NF-κB signaling as a significantly enriched pathway in AD subjects (FDR 1.92E-10), with both males and females sharing a feature of elevated levels of genes associated with the NF-κB signaling. Moreover, interaction networks functional enrichment analysis of the overall differentially expressed genes revealed a connection between two networks containing components of the NF-κB signaling pathway (*i.e.*, NFKB1, IKK1, NFKBIA) and astrocytes (i.e., GFAP, ALDH1L1, S100B) (Fig. 1b). NFKB1, the gene responsible for producing p50 NF-κB, a component of the NF-κB homodimer and heterodimer, positively correlated with GFAP (Fig. 1c) and ALDH1L1 (Fig. 1d) levels. The interaction networks functional enrichment analysis also revealed a connection between the NF-κB signaling pathway and microglia marker CX3CR1 (Supplementary Fig. 1c). However, NFKB1 and CX3CR1 levels did not directly correlate (Supplementary Fig. 1d).

To confirm the potential upregulation of NFKB1 in astrocytes, we analyzed data from two astrocyte-specific transcriptomic datasets. Mathys et al. included three groups: N (no AD pathology), EAD (mid-level AD pathology), and LAD (high-level AD pathology)^57^. Although astrocytic NFKB1 levels increased with disease severity, statistical significance was not reached (Fig. 1e). Similarly, NFKB1 levels were also slightly elevated in other cell types in AD, including microglia, inhibitory neurons, excitatory neurons, oligodendrocytes and oligodendrocyte precursor cells (OPCs) where its expression fluctuated with the course of disease progression (Supplementary Fig. 1e–i).

In Sadick’s study^58^, NFKB1 was significantly upregulated in astrocytes from AD subjects versus non-symptomatic (NS) patients in astrocyte clusters 0, 1, and 3 (Fig. 1f). Examples of the unique signature of these distinct astrocyte populations in AD include transcripts involved in synapse assembly, organization, and transmission in cluster 0, upregulation of transcripts involved in cell death and oxidative stress in cluster 1, and upregulation of transcripts involved in acute inflammatory responses in cluster 3. Since NF-κB modulates many of these responses ^19^, it will be interesting to determine whether this transcriptional factor plays a role in astrocyte diversity in future studies.

Lastly, we investigated the protein levels of p50 NF-κB and p-p65 NF-κB to validate the transcriptomic studies. Compared to non-AD subjects, we found higher p50 NF-κB (Fig. 1g,h) and p-p65 NF-κB (Fig. 1i,j) colocalization with clustered GFAP^+^-astrocytes in AD. Notably, our confocal colocalization studies suggested that clustered microglia-like cells also exhibit positivity for p-p65 NF-κB, as illustrated by the presence of p-p65 NF-κB staining in a microglia-like morphology in the representative panels in Fig. 1i. Although sparse, non-AD brains also presented clustered GFAP^+^-astrocytes and microglia-like cells positive to p-p65 NF-κB, which occurred due to the presence of lower but existent AD neuropathology.

These studies support the hypothesis that NF-κB contributes to astrocytes’ response to AD. Moreover, our data indicate that other CNS cells, including microglia, are likely associated with NF-κB-driven changes in AD, which warrants further investigation into these cells in future research.

### Chronic astrocytic NF-κB signaling accelerates neuronal senescence

Given that astrocytic NF-κB activation is a prominent feature of AD, we aimed to explore whether sustained activation of NF-κB at a young healthy age could trigger AD-like features such as persistent pro-inflammatory response and changes in neuronal molecular patterns and structure in a brain area associated with the disease, namely hippocampus. We used the ALDH1L1-Cre-ERT-IKK2CA mouse model, which enabled chronic activation of canonical NF-κB pathway through the conditional expression of a constitutively active IKK2 (IKK2CA), specifically in astrocytes^59,60^. Henceforth, vehicle- and tamoxifen-treated ALDH1L1-Cre-ERT-IKK2CA mice are described as A^IKK-OFF^ and A^IKK-ON^, respectively.

**Figure 2:**
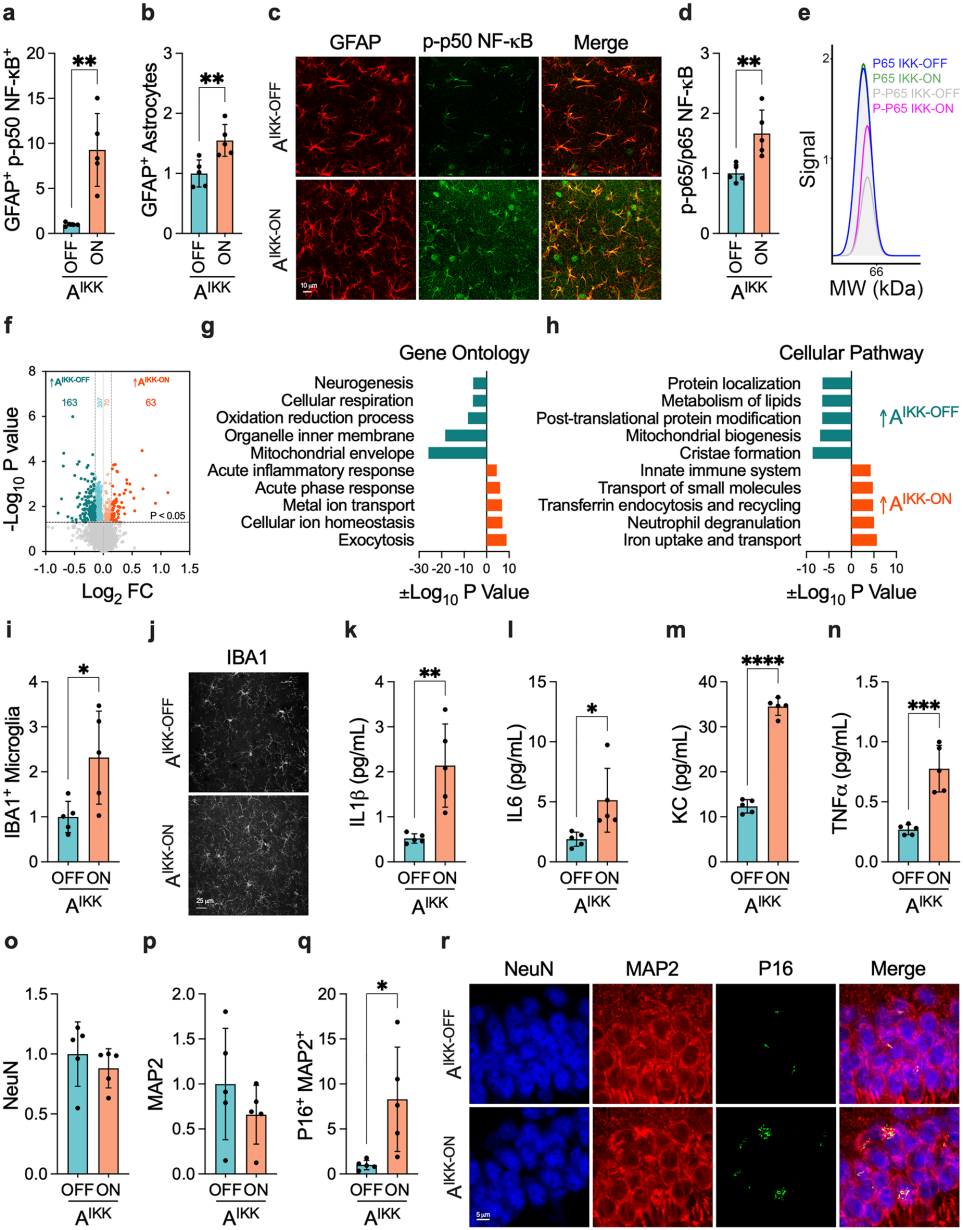
Changes in the healthy hippocampus caused by sustained astrocytic IKK2 activation. (**a**, **b**) Volumetric analysis of (**a**) p-p50 NF-κB in GFAP^+^-astrocytes (n = 5 per group, six 3D images per mouse) and (**b**) overall GFAP^+^-astrocytes (n = 5 per group, nine 3D images per mouse). (**c**) Illustrative images of p-p50 NF-κB and GFAP staining patterns. (**d**) Overall levels of p-p65 NF-κB normalized by total p65 NF-κB in hippocampal lysates (n = 5 per group). (**e**) Representative images of chemiluminescence signal detected at 65 kDa. (**f**) Volcano plot of targeted pairwise expression analysis between A^IKK-OFF^ and A^IKK-ON^ mice (n = 4–5 per group). (**g**, **h**) Differentially expressed proteins were analyzed using the GSEA software to determine the enrichment of (**g**) gene ontology and (**h**) cellular pathways terms. (**i**, **j**) Comparison of IBA1^+^-microglia between A^IKK-OFF^ and A^IKK-ON^ mice (n = 5 per group, three 3D images per mouse). (**k**–**n**) Levels of (**k**) IL1β, (**l**) IL6, (**m**) KC, and (**n**) TNFα in T-PER soluble hippocampal lysates (n = 5 per group). The levels of IFNγ, IL2, IL4, IL5, IL10, and IL12p70 were below the sensitivity of the multiplex kit. (**o**–**r**) Volumetric analysis of (**o**) NeuN (n = 5 per group, six 3D images per mouse), (**p**) MAP2 (n = 5 per group, six 3D images per mouse), and (**q**) P16^+^ MAP2^+^ (n = 5 per group, three 3D images per mouse) and (**r**) representative images in A^IKK-OFF^ and A^IKK-ON^ mice. Data were analyzed through an unpaired t-test. *P < 0.05; **P < 0.01; ***P < 0.001; ****P < 0.0001.

We first used confocal colocalization studies to validate the activation of astrocytic NF-κB after treating mice with tamoxifen. As expected, A^IKK-ON^ mice showed higher levels of p-p50 NF-κB versus A^IKK-OFF^ mice in the hippocampus (Fig. 2a,c). Likewise, GFAP levels were higher following NF-κB activation in astrocytes (Fig. 2b,c). Astrocytic IKK2 activation also led to an overall increase in the phosphorylation of p65 NF-κB in hippocampal lysates (Fig. 2d,e).

We applied unbiased proteomic analysis to identify changes in protein levels driven by the chronic activation of astrocytic IKK2 in the hippocampus (Fig. 2f–h, Supplementary Table 2) and cortex (Supplementary Fig. 2, Supplementary Table 3). We identified 63 upregulated and 163 downregulated proteins in the hippocampus of A^IKK-ON^ mice compared to A^IKK-OFF^ mice (Fig. 2f). By conducting in silico analysis using GSEA software^63,64^, we identified alterations in several gene ontologies (GO) (Fig. 2g) and cellular pathways (CP) (Fig. 2h). For instance, we found a loss of proteins associated with mitochondrial function and an increase of proteins related to the innate immune response in A^IKK-ON^ mice following chronic activation of astrocytic NF-κB. The cortex, which is another brain region mainly impacted by AD, exhibited comparable proteomic changes in A^IKK-ON^ mice (Supplementary Fig. 2).

Astrocytes are typically associated with a secondary inflammatory response after receiving signals from microglia. However, due to their expression of necessary biological components and proximity to the synaptic cleft, these cells are uniquely positioned to be the first to detect and signal changes associated with the buildup of AD-proteinaceous species. As a result, they may trigger the onset of microglia-mediated responses. Supporting this hypothesis, A^IKK-ON^ mice displayed higher intensity of IBA1^+^-microglia versus A^IKK-OFF^ mice (Fig. 2i,j). Likewise, hippocampal lysates from A^IKK-ON^ mice had higher IL1β, IL6, KC, and TNFα than A^IKK-OFF^ mice (Fig. 2k–n), indicating a heightened pro-inflammatory phenotype due to sustained activation of astrocytic NF-κB.

Finally, since chronic activation of astrocytic NF-κB resulted in the loss of mitochondrial proteins and an increase of immune-related proteins, we investigated whether these changes caused the ultimate debilitation of neuronal structure. NeuN (Fig. 2o,r) and MAP2 (Fig. 2p,r) levels in the hippocampus were comparable between A^IKK-ON^ and A^IKK-OFF^ mice, suggesting that the chronic activation of astrocytic NF-κB did not result in substantial loss of hippocampal neurons. However, higher levels of p16^INK4A^ (P16), a robust marker of cellular aging, were found in the hippocampal neurons of A^IKK-ON^ mice compared to A^IKK-OFF^ mice (Fig. 2q,r). Our studies indicate that the chronic activation of NF-κB in astrocytes is sufficient to incite molecular changes in the brain that culminate in neuronal senescence.

### Sustained inhibition of astrocytic NF-κB is associated with accelerated AD-like pathology

Next, our study delved into whether inhibition of astrocytic NF-κB could influence the progression of AD-like pathology in female 3xTgAD mice, a model that mirrors the accumulation of Aβ and tau seen in AD^61^. To achieve this, we overexpressed the inhibitor A20 under the control of the GFAP promoter using an AAV. First, we confirmed that the A20 overexpression was predominantly in hippocampal GFAP^+^-astrocytes in nTg mice (Supplementary Fig. 3a). While positive astrocytes were observed in adjacent brain areas, the pattern of expression was inconsistent. Hence, we focused solely on the hippocampus in the 3xTgAD mouse model. In nTg mice, A20 overexpression did not alter the colocalization of p-p65 NF-κB in GFAP^+^-astrocytes and the overall intensity of GFAP^+^-astrocytes and IBA1^+^-microglia in the hippocampus (Supplementary Fig. 3b–d). Furthermore, we could not determine cytokine levels, as signals in nTg hippocampal lysates were under the detection limit for all cytokines in the multiplex kit. A study involving aged nTg B6.129 mice has shown that despite the known involvement of astrocytes and NF-κB in CNS aging, 18-month-old mice did not display typical age-related changes such as long-term potentiation impairments or increased GFAP and IBA1 immunoreactivity, compared to 4-month-old mice^61^. Although the overexpression of A20 yielded suboptimal results in our nTg mice, further investigation of its effects in more refined models of aging astrocytes may be warranted.

**Figure 3:**
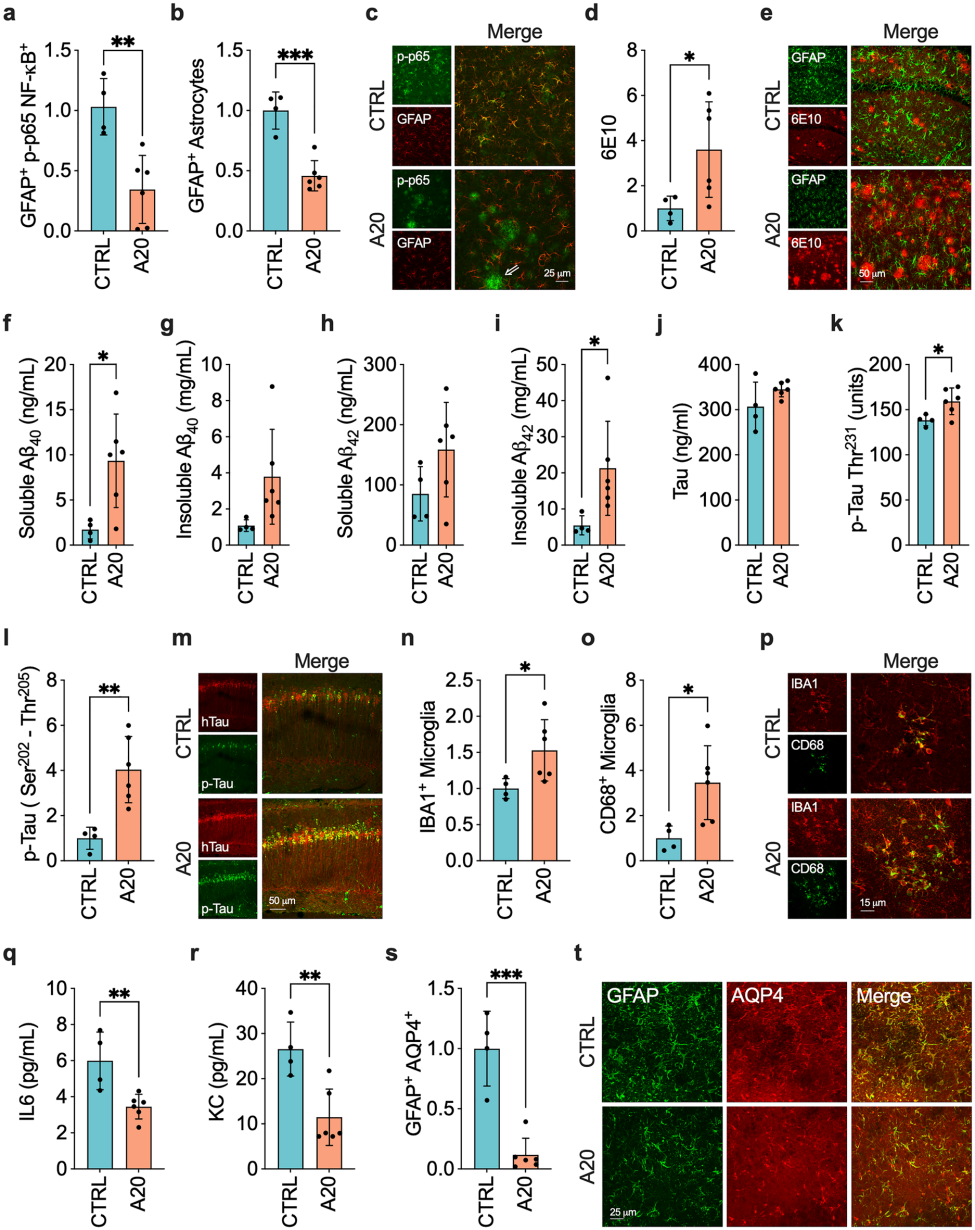
Changes in the hippocampus of 3xTgAD mice caused by overexpression of NF-κB inhibitor A20 in astrocytes. (**a**–**c**) Levels of (**a**) GFAP^+^-p-p65 NF-κB^+^ (n = 4–6 per group, three 3D images per mouse) and (**b**) GFAP^+^-astrocytes (n = 4–6 per group, nine 3D images per mouse) and (**c**) illustrative images. The arrow in the representative image indicates the presence of p-p65 NF-κB staining in clustered microglia-like cells. (**d**, **e**) 6E10^+^-Aβ plaques (n = 4–6 per group, nine 3D images per mouse) were higher in A20 expressing mice. (**f**–**i**) T-PER soluble and insoluble levels of Aβ peptides in hippocampal lysates (n = 4–6 per group). (**j**, **k**) Levels of (**j)** total and (**k**) phosphorylated-tau in T-PER soluble hippocampal lysates (n = 4–6 per group). (**l**) Levels of phosphorylated-tau and (**m**) representative images (n = 4–6 per group, three 3D images per mouse). (**n**–**p**) Levels of (n) IBA1^+^-microglia (n = 4–6 per group, nine 3D images per mouse) and (**o**) CD68^+^-microglia (n = 4–6 per group, six 3D images per mouse) and (p) representative images. (**q**, **r**) Levels of (**q**) IL6 and (**r**) KC in hippocampal lysates (n = 4–6 per group). Inhibition of astrocytic NF-κB did not alter IL1β, IL4, IL5, and TNFα levels. IFNγ, IL2, and IL12p70 levels were below the sensitivity of the multiplex kit. (**s**) Quantification of AQP4^+^-GFAP^+^ colocalization (n = 4–6 per group, three 3D images per mouse) and (**t**) representative images. Data were analyzed through an unpaired t-test. *P < 0.05; **P < 0.01; ***P < 0.001.

In the 3xTgAD mouse model, confocal colocalization studies confirmed the upregulation of A20 (Supplementary Fig. 4a,b) and reduction of p-p65 NF-κB (Fig. 3a,c) in GFAP^+^-astrocytes in the hippocampus of AAV-GFAP-A20-treated mice. A20 overexpression correlated with lower detection of GFAP^+^-astrocytes (Fig. 3b,c). Conversely, capillary-based immunoassay results indicated slightly decreased p-p65 levels in bulk hippocampal lysate due to A20 overexpression, although the reduction was not statistically significant compared to AAV control (Supplementary Fig. 4c–e). Considering that NF-κB phosphorylation occurs in various CNS cells that can be independently affected by AD-like pathology, the lack of significant change in bulk p-p65 levels due to the astrocyte-specific A20 intervention is plausible. The confocal microscopy images revealed numerous p-p65^+^ neuronal- and microglial-like cells, predominantly clustered microglia-like cells, as depicted in the representative panels in Fig. 3c. We opted not to explore the association of NF-κB with cells other than astrocytes as it falls beyond the scope of our study.

We then focused on whether inhibition of astrocytic NF-κB could mitigate AD-like pathology. Notably, A20 overexpression resulted in higher levels of Aβ peptides (Fig. 3d–i). Likewise, mice treated with AAV-GFAP-A20 exhibited increased levels of phosphorylated-tau (Fig. 3k–m), while total tau (Fig. 3j) remained unchanged compared to AAV-CTRL-treated mice. To elucidate the mechanism responsible for the increase in Aβ, we determined the impact of AAV-GFAP-A20 treatment on APP processing. We used the anti-APP C-Terminal (751–770) antibody, which recognizes full-length APP and its proteolytic fragments c-terminal APP fragment (CTF)β [a 99-amino acid-long CTF produced by β-secretase cleavage (C99)] and CTFα [an 83-amino acid–long C-terminal APP fragment produced by α-secretase cleavage (C83)]. The inhibition of astrocytic NF-κB did not alter APP steady-state levels nor the levels of its CFT fragments, indicating that A20 overexpression did not change Aβ production (Supplementary Fig. 4f–h).

We next investigated microglia response since it has been shown that astrocytic NF-κB facilitates the removal of Aβ through a process linked with the microglia polarization towards a phagocytic phenotype^54^. However, in our study, the intensity of plaque-associated IBA1^+^-microglia and the levels of phagocytic marker CD68 in these cells were higher in animals treated with AAV-GFAP-A20 (Fig. 3n–p). The elevated microglial response is linked to higher Aβ levels, indicating that at the late disease stage, microglia react to Aβ regardless of astrocyte function and trigger a matching phagocytic response.

Finally, we determined the levels of molecules closely linked with the reactive phenotype of astrocytes in AD. Inhibition of NF-κB in astrocytes resulted in lower IL6 and KC steady-state levels, molecules regulated by NF-κB commonly enriched in astrocytes^65,66^ (Fig. 3q,r). We also investigated a disease-modifying mediator regulated by NF-κB and highly expressed in astrocytes. It has been shown that NF-κB modulates the expression of AQP4, a key mediator of the glymphatic system linked with the clearance of Aβ and tau^7,30,67–69^. Notably, A20 overexpression reduced AQP4 colocalization with GFAP^+^-astrocytes (Fig. 3s,t) and the overall AQP4 levels in hippocampal lysates (Supplementary Fig. 4i–k).

Altogether, we found that the NF-κB signaling pathway regulates the expression of multiple molecules commonly associated with the neurotoxic pro-inflammatory phenotype of astrocytes (*i.e.*, high GFAP, IL6, and KC) and with the neuroprotective phenotype of these cells (*i.e.*, AQP4) in a mouse model of AD.

## Discussion

Our study indicates that the NF-κB signaling pathway is chronically associated with astrocyte response in AD, and it plays an essential role in the response of these cells in healthy and diseased brains. Specifically, we found an enrichment of NFKB1 (p50 NF-κB) and p-p65 NF-κB in astrocytes in AD subjects. Molecular studies in mice showed that chronic activation of astrocytic NF-κB drives proteomic changes in the healthy brain, including the increase in inflammatory-related proteins and loss of mitochondria-associated proteins, which ultimately have the potential to accelerate the senescence of neurons. We also found that astrocytic NF-κB is required to upregulate proteins generally associated with these cells’ neurotoxic and pro-inflammatory phenotype in AD. Intriguingly, we discovered that a certain level of astrocytic NF-κB activity is required to maintain some of the neuroprotective features of these cells in AD since its inhibition was associated with reduced expression of the disease-modifying mediator AQP4 as well as higher accumulation of Aβ and tau in mice.

NF-κB possesses biological characteristics that make it a significant player in shaping astrocyte plasticity. Its capability to modulate the expression of various genes allows it to influence cell responses to physiological and pathological stimuli. Therefore, it is imperative to regulate the activity of this transcriptional factor with great precision. In this regard, we were initially interested in determining whether sustained activation of astrocytic NF-κB is sufficient to promote substantial effects in the healthy brain, leading to molecular and cellular changes featured in AD. To this goal, we engineered a conditional tamoxifen-induced mouse model to chronically activate IKK2 (upstream activator of NF-κB) under the control of the astrocyte-enriched ALDH1L1 promoter^59,60^. Notably, other research groups have used a strategy complementary to the one we applied to induce the activation of IKK2 in astrocytes. These studies used the GFAP promoter conditionally regulated by tetracycline to modulate the IKK2CA transgene (GFAP-tTA-IKK2-CA mice). Both strategies have strengths and limitations. For instance, ALDH1L1 is expressed in all astrocytes, whereas GFAP does not identify all astrocytes throughout the CNS. Both models can exhibit distinct off-target effects that might act as confounders because neural progenitor cells, nascent neurons, and type 1 neural stem cells express GFAP, while some cells from peripheral organs express ALDH1L1^70^. Therefore, comparing the effects of ALDH1L1- and GFAP-induced activation of astrocytic IKK2 helps overcome their constraints. It also offers a unique opportunity to narrow down co-occurring mechanisms linked with chronic astrocytic NF-κB signaling.

A well-defined overlapping characteristic in all studies using the activated astrocytic IKK2 is the exacerbated inflammatory response in multiple CNS regions, such as the hippocampus, cortex, substantia nigra, cerebellum, and spinal cord. Activation of NF-κB signaling in astrocytes triggered changes in the levels of inflammatory mediators (*e.g.*, cytokines) and markers of astrocytes (*i.e.*, GFAP) and microglia (*i.e.*, IBA1, CD45, CD11b, CD68)^27,54,71–73^. Notably, some molecular changes induced by sustained NF-κB signaling in astrocytes were equivalent to changes rendered in mouse models of amyotrophic lateral sclerosis (ALS)^71^ and Parkinson's disease (PD)^73^.

Loss-of-function mouse models further support the relevance of astrocytic NF-κB in inflammatory responses and disease pathogenesis. Multiple studies have shown that selective knockout of components of the IKK complex in astrocytes reduces inflammation and neurodegenerative severity in mouse models of glaucoma^23^, PD^24^, autoimmune encephalomyelitis^29^, and high-fat diet-induced obesity^74^. Likewise, selective inactivation of astrocytic NF-κB by expressing a dominant negative form of IκBα under the control of the GFAP promoter inhibited inflammation and improved functional recovery in mouse models of experimental autoimmune encephalomyelitis^28^, spinal cord injury^75,76^, pain^77,78^, ischemic injury^79^, sciatic nerve injury^80^, retinal ischemia–reperfusion injury^81^, and experimental optic neuritis^82^. Transgenic inhibition of astrocytic NF-kB signaling also ameliorated gliosis and axonal loss, maintained white matter structural integrity, and preserved memory function in a mouse model of vascular dementia^25^.

Our unbiased proteomic studies validated these previous findings since chronic activation of IKK2 in astrocytes increased proteins associated with the innate immune system. Similarly, we found higher levels of GFAP^+^-astrocytes and IBA1^+^-microglia and increased levels of the pro-inflammatory mediators IL1β, IL6, KC, and TNFα. Importantly, we discovered that sustained astrocytic NF-κB signaling leads to proteomic changes beyond the intensification in inflammation, at least in the hippocampus and cortex. Remarkably, a more significant proportion of proteins were downregulated in A^IKK-ON^ mice, suggesting the impairment of vital cellular processes like mitochondrial biogenesis, cellular respiration, and metabolism of lipids. Finally, we established an acceleration of neuronal senescence, as indicated by the higher levels of protein p16^INK4A^ in neurons following the activation of IKK2 in astrocytes.

Additional studies are needed to uncover the missing pieces linking the molecular events occurring following the activation of astrocytic NF-κB. Nonetheless, its continued signaling in astrocytes likely leads to an initial buildup of immune-related proteins in the hippocampus, cortex, and other CNS domains. Because proper astrocytic NF-κB-mediated responses must be tightly regulated and limited, the accumulation and perpetuation of inflammatory signals then drive unintended neurotoxic outcomes, such as mitochondrial impairment, neuronal senescence, and neurodegenerative-like phenotypes (*i.e.*, PD, ALS)^83^. This hypothesis agrees with studies showing the association among dysregulated NF-κB signaling, inflammation, cellular senescence, and aging^84–90^.

All these studies lead to the assumption that inhibition of NF-κB in astrocytes is a promising alternative to mitigate the neuropathological process associated with AD. Indeed, an extensive array of cell culture studies have supported this claim by demonstrating that Aβ- and tau-induced activation of NF-κB in astrocytes results in the upregulation of pro-inflammatory mediators and release of neurotoxic factors^42,48,51,52,91–93^. Moreover, astrocytic NF-κB drives the expression of mediators with potent neurotoxic properties in AD. Complement C3 is a powerful example of a NF-κB-regulated signal in astrocytes extensively characterized as a neurotoxic mediator in AD mouse models, and its inhibition mitigates disease pathology^52,94–97^.

However, our studies show that the impact of astrocytic NF-κB in AD is not straightforward. Using an AAV approach to overexpress the NF-κB inhibitory protein A20 in GFAP^+^-astrocytes, we found that the accumulation of pathological forms of Aβ and tau accelerates upon the blockage of astrocytic NF-κB signaling. First, we found that APP processing was not altered by the expression of astrocytic A20, as indicated by unaltered levels of APP and its CTF fragments. Since astrocytes can communicate with microglia, we also evaluated whether microglia phagocytic function was impaired. Notably, it has been shown that activation of astrocytic NF-κB facilitates the clearance of Aβ through a process linked with microglia polarization towards a phagocytic phenotype^54^. However, in our study, overall levels of IBA1 and CD68 were augmented in animals treated with AAV-GFAP-A20, indicating that microglia function increased accordingly with the buildup of AD pathology. This discrepancy in the mechanisms of action of NF-κB in astrocytes is likely related to timing in the disease process. In Yang’s study, astrocytic NF-κB activity was triggered in young mice before the buildup of the disease ^54^. In our model, however, inhibition of NF-κB occurred later in life when animals already displayed substantial levels of AD markers. Noteworthy, our study agrees with Kraft and colleagues, who have shown that genetic attenuation of astrocyte reactivity to AD-like pathology in mice is associated with higher Aβ deposition and abundance of microglia around plaques^98^. These studies emphasize that NF-κB likely regulates astrocytes' extraordinary plasticity and that astrocytes have a significant role in the clearance of AD proteinaceous forms.

Previous studies have demonstrated that ablation of astrocyte reactivity in AD mouse models increased monomeric Aβ production and reduced degradation of Aβ, heightened expression of pro-inflammatory cytokines, decreased expression of synaptic markers, and exacerbated cognitive deficits^98–100^, supporting the fundamental role of these cells in counterbalancing the disease process. In particular, the deletion of GFAP and vimentin in the APP/PS1 mouse model of AD increased the Aβ load and the number of dystrophic neurites, reinforcing the prominent role of astrocytes in limiting plaque growth and attenuating plaque-related dystrophic neurites^98^. Hence, we focused on NF-κB-regulated disease-associated molecules highly expressed in astrocytes to reveal underlying molecular mechanisms driving the acceleration of AD-like pathology in the 3xTgAD mouse model. Our data showed that A20-driven inhibition of NF-κB leads to suppressing astrocyte reactivity in response to AD-like pathology, as evidenced by the lower levels of multiple proteins commonly upregulated in astrocytes in AD, such as GFAP, IL6, and KC^65,66,101^. We also demonstrated that NF-κB activity is required to regulate AQP4 levels in GFAP^+^-astrocytes in the 3xTgAD mouse model. AQP4 is a water channel expressed on astrocytic end feet in the brain and associated with the clearance of proteins through the glymphatic system. In AD, failure of AQP4 activity resulted in the buildup of Aβ and tau^7,30,67–69^. Therefore, changes in NF-κB activity in astrocytes did not alter a singular pathway but the function of multiple genes that cooperate to regulate the homeostatic function of these cells. Although chronic activation of NF-κB is linked to astrocytes' neurotoxic and pro-inflammatory phenotype, the sustained inhibition of NF-κB signaling suppresses astrocyte plasticity, effectively hindering its capacity to react appropriately to the changes in AD.

Research in the field has only begun to delve into the intricate relationship among NF-κB, astrocytes, and AD. The complexity of this association necessitates further investigations in both healthy and disease conditions. These investigations can advance our understanding of underlying molecular mechanisms and potentially lead to novel therapeutic approaches. Thus far, efforts to identify signals that regulate astrocytes' protective and neurotoxic roles in the CNS have revealed that numerous signaling pathways have overlapping functions in physiological and pathological conditions. Although challenging, we can pinpoint targets that may significantly impact diseases by dissecting how specific cellular pathways and mediators operate in distinct situations and biological systems. In this regard, our study offered new insights into how disruptions to astrocytic NF-κB signaling can adversely affect brain function in both health and AD. While completely blocking astrocytic NF-κB signaling may not be a feasible therapeutic approach due to the diversity of associated processes, restoring downstream effects such as complement C3, AQP4, and mitochondrial function could significantly enhance the management of AD.

### Supplementary Information

Supplementary Figures.

Supplementary Table 1.

Supplementary Table 2.

Supplementary Table 3.

## Data Availability

The data supporting this study's findings are available from the corresponding author upon reasonable request.
