# Supplementary material for: The impact of astrocytic NF-κB on healthy and Alzheimer’s disease brains
Source: Sci Rep. 2024 Jun 21;14:14305. doi: 10.1038/s41598-024-65248-1 (PMC11192733; doi:10.1038/s41598-024-65248-1)
Supplement: Supplementary file 1 — Supplementary Figures. [file 41598_2024_65248_MOESM1_ESM.pdf]

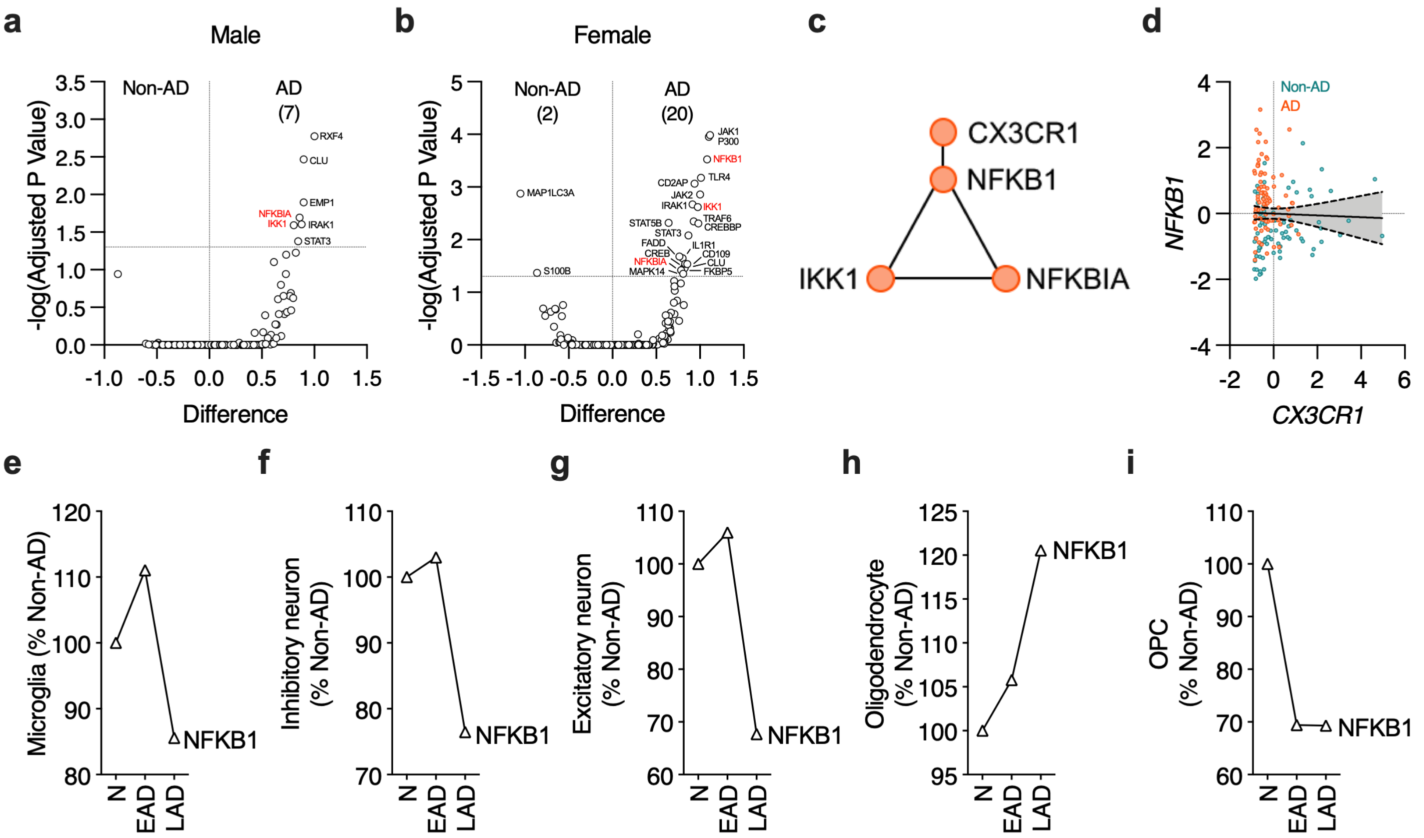

Supplementary Figure 1 – Association of NF-κB with CNS cells. (a, b) Normalized transcriptomic data from the temporal cortex of (a) males and (b) females was obtained from the MayoPilot RNAseq study 55. The data comprises information from 78 non-AD subjects (37 female and 41 male) and 82 AD subjects (49 female and 33 male). Differentially expressed genes were determined by unpaired t-test followed by Holm-Sidak's multiple comparisons tests. The accepted level of significance for the tests was $P < 0.05$. The volcano plot displays the differences in Z-scores between AD and non-AD subjects. (c) Interaction networks functional enrichment analysis of the differentially expressed genes in the STRING database revealed a connection between the NF-κB signaling pathway and microglia marker CX3CR1. (d) The correlation between NFKB1 and CX3CR1 was determined using linear regression and Pearson correlation. (e-i) NFKB1 levels in (e) microglia, (f) inhibitory neurons, (g) excitatory neurons, (h) oligodendrocytes and (i) OPCs isolated from individuals with no AD pathology (N, n = 24), mid-level AD pathology (EAD, n = 15), and high-level AD pathology (LAD, n = 9) obtained from Mathys' study 57. We represented the data relative to the mean level of the no pathology group.

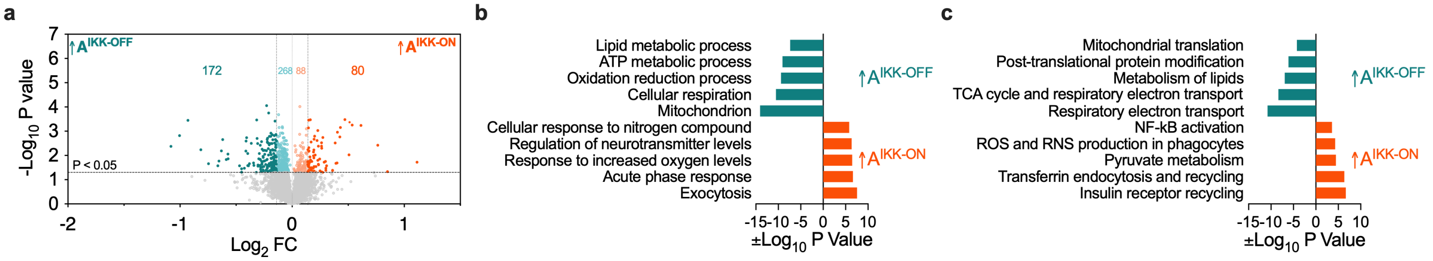

Supplementary Figure 2 – Proteomic analysis of TCX lysates from AIKK-OFF and AIKK-ON mice. (a) Volcano plot of targeted pairwise expression analysis. (b, c) Differentially expressed proteins were analyzed using the GSEA software to determine the enrichment of (b) gene ontology and (c) cellular pathways terms.

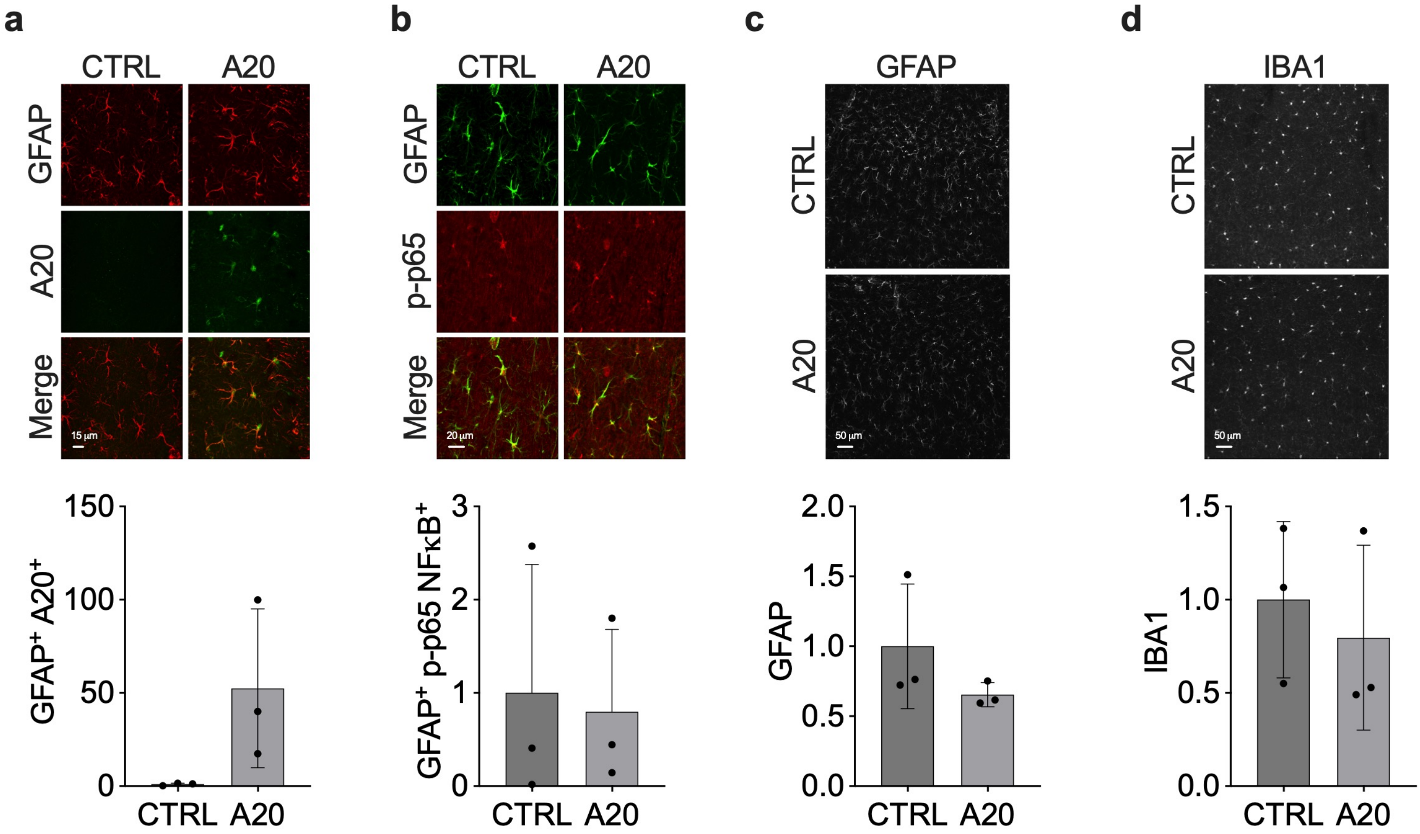

Supplementary Figure 3 – Characterization of AAV-GFAP-A20 in the hippocampus of nTg mice. Illustrative images and quantification of (a) A20 and (b) p-p65 NF-κB immunoreactivity in $GFAP^{+}$-astrocytes, and overall (c) GFAP and (d) IBA1 levels (n = 3 per group, four 3D images per mouse).

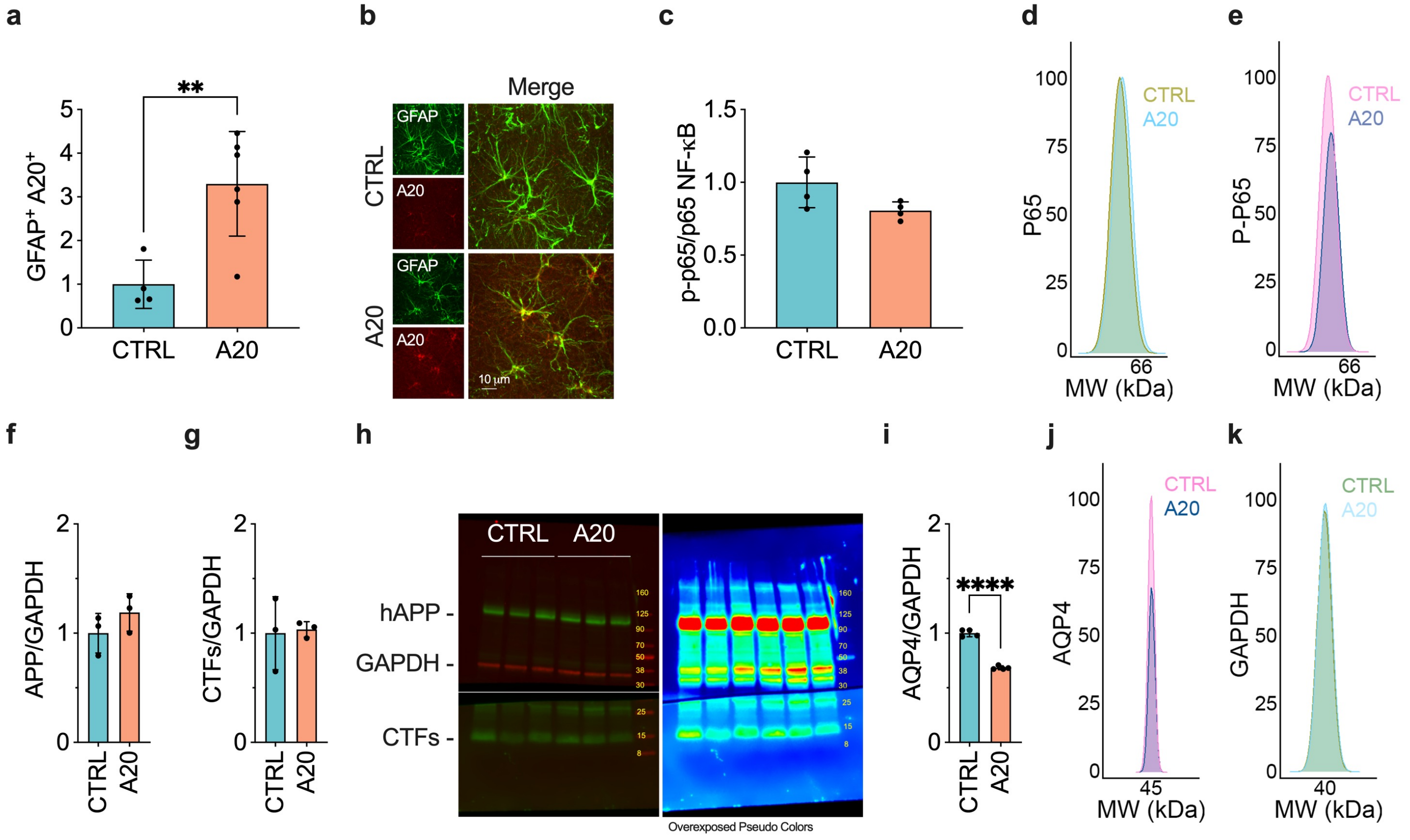

Supplementary Figure 4 – Changes in the hippocampus of 3xTgAD mice caused by overexpression of NF-κB inhibitor A20 in astrocytes. (a, b) Levels of (a) GFAP$^+$-A20$^+$ (n = 4-6 per group, four 3D images per mouse) and (b) illustrative images. (c) Levels of p-p65 NF-κB were normalized by the total levels of p65 NF-κB in hippocampal lysates of 3xTgAD mice (n = 4 per group). (d, e) Representative images of chemiluminescence curves detected at 65 kDa for (d) total p65 NF-κB and (e) p-p65 NF-κB. (f, g) A20 overexpression did not change levels of (f) APP and (g) its C-terminal fragments (CTFs) in hippocampal lysates (n = 3 per group). (h) The left panel shows an original unprocessed APP-CT20 (green channel) and GAPDH (red channel) staining. All samples were run in the same gel and transferred to the same membrane. We cut the membrane at ~ 30 kDa before exposing it to anti-APP-CT20 and GAPDH antibodies to maximize the detection of lower CTFs (~ ten kDa). The right panel shows overexposed pseudo color detection to show the cut line of the upper and lower parts of the membrane displaying full-length blots and membrane edges. (i) GAPDH levels normalized levels of AQP4 in hippocampal lysates of 3xTgAD mice (n = 4 per group). (j, k) Representative images of chemiluminescence curves detected for (j) AQP4 and (k) GAPDH.
